# Kynurenines and the Endocannabinoid System in Schizophrenia: Common Points and Potential Interactions

**DOI:** 10.3390/molecules24203709

**Published:** 2019-10-15

**Authors:** Ferenc Zádor, Gábor Nagy-Grócz, Gabriella Kekesi, Szabolcs Dvorácskó, Edina Szűcs, Csaba Tömböly, Gyongyi Horvath, Sándor Benyhe, László Vécsei

**Affiliations:** 1 Institute of Biochemistry, Biological Research Center, Temesvári krt. 62., H-6726 Szeged, Hungary; zador.ferenc@gmail.com (F.Z.); dvoracsko.szabolcs@brc.mta.hu (S.D.); szucsedina7@gmail.com (E.S.); tomboly.csaba@brc.mta.hu (C.T.); benyhe.sandor@brc.mta.hu (S.B.); 2 Faculty of Health Sciences and Social Studies, University of Szeged, Temesvári krt. 31., H-6726 Szeged, Hungary; gabor.balazs.nagy@gmail.com; 3 Department of Neurology, Faculty of Medicine, Albert Szent-Györgyi Clinical Center, University of Szeged, Semmelweis u. 6., H-6725 Szeged, Hungary; 4 Department of Physiology, Faculty of Medicine, University of Szeged, Dóm tér 10., H-6720 Szeged, Hungary; kekesi.gabriella@med.u-szeged.hu (G.K.); horvath.gyongyi@med.u-szeged.hu (G.H.); 5 Doctoral School of Theoretical Medicine, Faculty of Medicine, University of Szeged, Dóm tér 10., H-6720 Szeged, Hungary; 6 Interdisciplinary Excellence Center, Department of Neurology, University of Szeged, Semmelweis u. 6., H-6725 Szeged, Hungary

**Keywords:** cannabinoids, endocannabinoids, cannabinoid receptors, kynurenines, kynurenine pathway, schizophrenia

## Abstract

Schizophrenia, which affects around 1% of the world’s population, has been described as a complex set of symptoms triggered by multiple factors. However, the exact background mechanisms remain to be explored, whereas therapeutic agents with excellent effectivity and safety profiles have yet to be developed. Kynurenines and the endocannabinoid system (ECS) play significant roles in both the development and manifestation of schizophrenia, which have been extensively studied and reviewed previously. Accordingly, kynurenines and the ECS share multiple features and mechanisms in schizophrenia, which have yet to be reviewed. Thus, the present study focuses on the main common points and potential interactions between kynurenines and the ECS in schizophrenia, which include (i) the regulation of glutamatergic/dopaminergic/γ-aminobutyric acidergic neurotransmission, (ii) their presence in astrocytes, and (iii) their role in inflammatory mechanisms. Additionally, promising pharmaceutical approaches involving the kynurenine pathway and the ECS will be reviewed herein.

## 1. Introduction

Schizophrenia, which is among the major psychiatric syndromes, affects approximately 1% of the population worldwide. The combined economic and social costs associated with this disorder rank it as the 15th highest cause of disease-related disabilities worldwide [1]. Schizophrenia is characterized by positive symptoms (i.e., hallucination, delusions, confused thought, and disorganized speech), negative symptoms (i.e., asocial behavior, blunted emotions and motivation, affective flattening, alogia, and avolition), and cognitive dysfunctions. Currently used antipsychotic medications have displayed insufficient efficacy and are mostly restricted to the improvement of positive symptoms, given their limited or no effect on negative symptoms and cognitive impairments. Although the exact pathophysiology of schizophrenia still remains unknown, certain theories have emerged, which involve, for instance, the dopaminergic and glutamatergic systems [2]. Recently, the endocannabinoid system (ECS) and kynurenic acid (KYNA) hypotheses—an extension of the glutamatergic dysfunction model—have gained attention.

KYNA, kynurenines, and their associated elements (see Section 2.1.) share several physiological functions with the ECS (see Section 3.1.). Furthermore, both systems are similarly dysfunctional in schizophrenia [3,4]. This has led to the assumption of their interaction, which could be utilized for therapeutic applications. This concept has been recently discussed by us [5] and others [6] in reviews.

**Figure 1:**
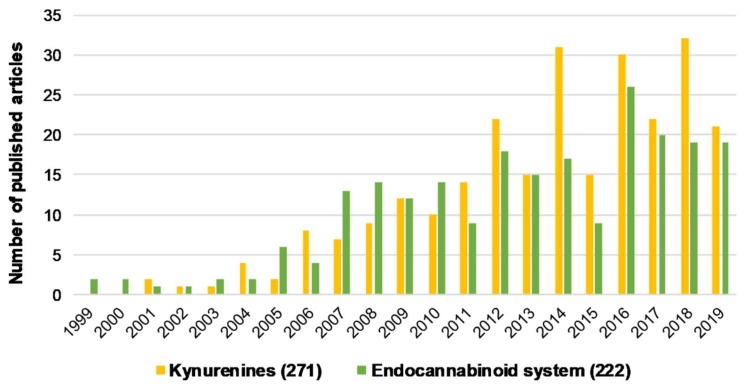
The number of articles published regarding kynurenines and the endocannabinoid system individually associated with schizophrenia from the last 20 years. Brackets indicate the total number of publications from the last 20 years. Data was obtained from PubMed using “kynurenines AND schizophrenia” and “endocannabinoid system AND schizophrenia” as keywords.

Both kynurenines and the ECS have been separately implicated in schizophrenia and discussed previously in numerous publications (Figure 1, Table 1). However, their common points and potential interactions relevant to schizophrenia have yet to be reviewed. Thus, the present review aims to gather and highlight related data and draw attention to potential interactions that might help us better understand the pathology/etiology of schizophrenia. Although data describing the direct interaction between the two systems in schizophrenia may be missing in some cases, multiple overlapping functions/alterations in the two systems indicate the possibility of an interaction. Accordingly, such potential interactions will be the focus of this review. To obtain a better overview of these points, this review will cover a separate general introduction to kynurenines and the ECS. Additionally, possible hypotheses for the mechanism of schizophrenia related to this review will be discussed in the appropriate sections (see Section 2.2., Section 3.2., Section 4.2.1., and Section 4.4.1.). Finally, new potential drug targets for both systems will also be discussed (see Table 1).

## 2. Kynurenines and Their Role in Schizophrenia

### 2.1. Kynurenines and Associated Elements

#### 2.1.1. The Kynurenine Pathway

**Figure 2:**
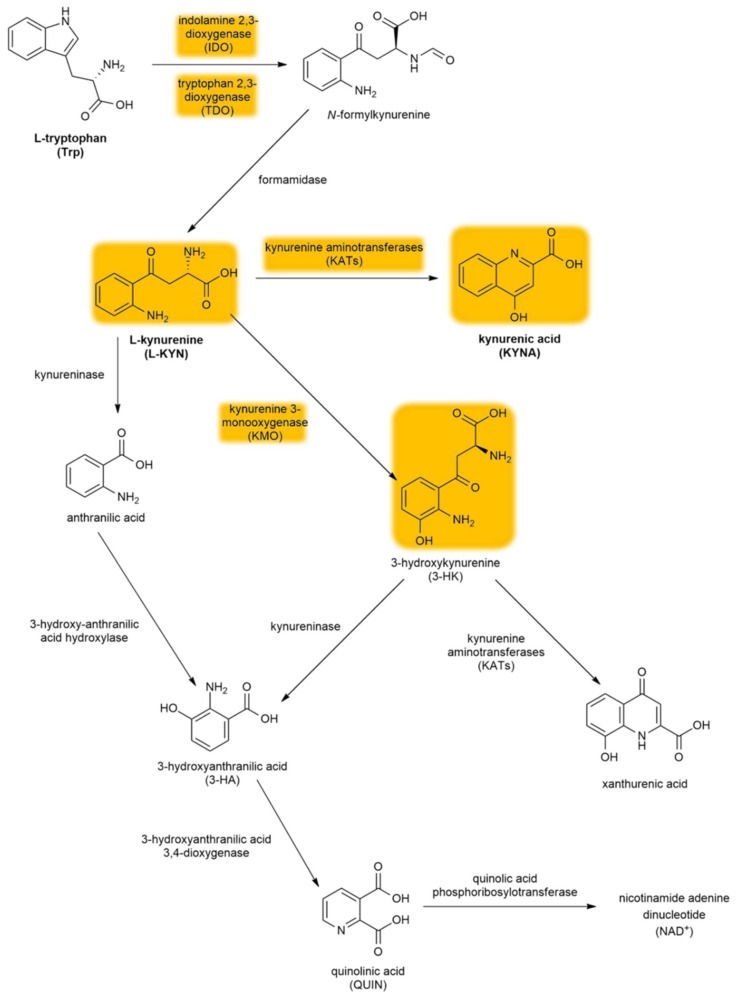
The kynurenine pathway. The yellow background indicates the metabolites and enzymes relevant to schizophrenia. Abbreviations of metabolites and enzymes frequently used in the text are also indicated.

The kynurenine pathway (KP) is a collection of metabolic substances and enzymes present in the synthesis and degradation of l-kynurenine (l-KYN). This process is the main metabolic route of tryptophan (Trp) (Figure 2). The initial and rate-limiting step in the KP consists of two iron-dependent enzymes, indoleamine 2,3-dioxygenase 1 and 2 (IDO1 and IDO2) and tryptophan 2,3-dioxygenase (TDO). These enzymes embed molecular oxygen through the 2–3 bond of the Trp indole moiety. IDO is a monomer found in the central nervous system (CNS), whereas TDO is a homotetramer having stiff substrate selectivity, and it occurs primarily in peripheral tissues, especially in hepatic tissue. IDO and TDO catalyzes Trp to *N*-formyl-l-kynurenine by opening the Trp ring and further hydrolyze it to l-KYN by formamidase. l-KYN can cross the blood–brain barrier completely and exert neuroprotective effects. Roughly 60% of l-KYN present in the CNS is absorbed from the blood by glial cells.

l-KYN can be converted via three different pathways. The first metabolic route involves the conversion of l-KYN into anthranilic acid by kynureninase and further into 3-hydroxyanthranilic acid (3-HA) by 3-hydroxy-anthranilic acid 3,4-dioxygenase. The second branch of the KP begins with the hydroxylation of l-KYN at the third position by kynurenine 3-monooxygenase (KMO), which produces 3-hydroxykynurenine (3-HK) that can be further converted into xanthurenic acid and 3-HA. Notably, anthranilic acid can also be converted into 3-HA, which can be further converted into pyridine-2,3-dicarboxylic acid or quinolinic acid (QUIN), which is an *N*-methyl-d-aspartate receptor (NMDAR) agonist that causes lipid peroxidation [31]. In the final step of this KP branch, QUIN is then degraded into nicotinamide adenine dinucleotide (NAD+) [32]. The last branch of the KP starts with the conversion of l-KYN into KYNA by kynurenine aminotransferases (KATs), which have four subtypes with various biochemical profiles [33]. In contrast to QUIN, KYNA is an endogenous glutamate receptor antagonist. Under physiological conditions, the KAT II enzyme is responsible for the biosynthesis of KYNA in the brain [34]. KATs are chiefly present in astrocytes [35] (see Section 4.3.2.), unlike other enzymes (e.g., KMO) that are primarily expressed in microglia [36].

#### 2.1.2. KYNA and Its Target Receptors

In 1853, KYNA was first discovered in dog urine by a German chemist, Justus von Liebig. After 50 years, Ellinger and Homer revealed that KYNA is produced during Trp metabolism. This metabolic route for Trp was first described in 1947 in a process called the KP [37]. Almost all KP metabolites have a broad spectrum of biological effects and have been associated with numerous disorders [38], such as multiple sclerosis [39], Parkinson’s disease [40], migraine [41], and schizophrenia [2], which will be further discussed.

KYNA can influence different types of receptors. Accordingly, it behaves as an antagonist at the strychnine-insensitive glycine-binding site of NMDARs at low concentrations [42], while also blocking the glutamate-binding site of NMDARs at higher doses [43]. Moreover, KYNA causes weak antagonistic effects on kainate- and α-amino-3-hydroxy-5-methyl-4-isoxazolepropionic acid (AMPA)-sensitive glutamate receptors [42], with its impact on AMPA receptor-mediated action being concentration dependent. This effect is facilitatory at low concentrations (nanomolar to micromolar) and inhibitory at high concentrations (micromolar to millimolar) [44]. Although published data have suggested that KYNA also functions as an α7 nicotinic acetylcholine receptor (α7nAChR) antagonist [45] by reducing the presynaptic release of glutamate, this concept is currently under debate [46]. Another review reported that KYNA can be considered a bona fide endogenous modulator for α7nAChR, although it is a complex phenomenon that depends mostly on methodological considerations [47]. Furthermore, KYNA has an agonistic effect on the G protein-coupled receptor 35 (GPR35) [48,49], as well as on the aryl hydrocarbon receptors (AHR) [50]. Our group previously demonstrated that KYNA displays diverse effects depending on its concentration (few hundred nanomolar vs. micromolar), possibly through different receptor targets [51,52,53].

### 2.2. The KYNA Hypothesis of Schizophrenia

The KYNA hypothesis of schizophrenia has been studied and reviewed previously by numerous authors [2,4,26,54,55]. This section will briefly discuss the background of this hypothesis, which is based on the finding that exogenous NMDAR antagonists—such as phencyclidine and ketamine—induce schizophrenia-like symptoms that can be mimicked by KYNA [4,7,56,57,58]. The hypothesis is also supported by clinical data, given that patients with schizophrenia show increased KYNA levels in the prefrontal cortex (PFC) (2.9 pmol/mg protein vs. 1.9 pmol/mg protein) [59] and cerebrospinal fluid (CSF) (~1.7 vs. 1 nM) [60]. According to preclinical data, this elevation can lead to behavioral and neurotransmission changes associated with schizophrenia, such as cognitive deficits and disrupted glutamatergic, γ-aminobutyric acidergic (GABAergic), cholinergic, and dopaminergic signaling [56,61,62,63,64,65,66,67,68,69]. Additionally, the inhibition of KYNA formation has been found to improve such symptoms [70] (see Section 5.3.). The increase in KYNA levels in schizophrenia is partly due to the altered enzyme activity/expression in the KP, which shifts Trp metabolism to KYNA production [8]. KYN levels in the CSF and cortical brain regions are also increased in patients with schizophrenia [71,72], whereas the neurotoxic branch of the KP (QUIN, 3-HK) seems to be unaffected [59,73,74]. Additionally, studies have found reduced expression of KYNA target receptors, namely, NMDAR [75] and α7nAChR, in postmortem brain samples of patients with schizophrenia [76,77].

## 3. The Endocannabinoid System and Its Role in Schizophrenia

### 3.1. Overview of the Endocannabinoid System

The ECS, which mainly consists of two well-characterized receptors, primarily endogenous lipid-derived ligands called endocannabinoids, and enzymes responsible for their synthesis and degradation, is involved in various physiological and pathological processes of the CNS and certain peripheral organs [19,78].

To date, two types of cannabinoid receptors, cannabinoid receptor type 1 (CB_1_R) and cannabinoid receptor type 2 (CB_2_R), belonging to the family of G_i/o_ protein-coupled receptors (GPCRs) have been cloned [79,80,81]. Accordingly, their activation inhibits cAMP production, stimulates mitogen-activated protein kinases, and presynaptically suppresses the release of several neurotransmitters relevant to schizophrenia (see Section 4.2.3.) [10,82,83]. CB_1_Rs play a role in regulating mood or emotions, antinociception, energy balance, immune mechanisms, and endocrine functions [19,84]. Although CB_1_Rs are located predominantly in the hippocampus, basal ganglia, cortex, amygdala, and cerebellum, they are also highly expressed in the liver, adipose tissues, muscles, cardiovascular system, and gastrointestinal system (GI) [19,85]. Additionally, CB_1_Rs are known to be the most abundantly expressed GPCR in the CNS [86,87]. On the other hand, CB_2_Rs are expressed predominantly in immune and hematopoietic cells, although they can also be found in the CNS, such as in microglia [88]. Generally, CB_2_Rs have a protective role, they reduce inflammation-induced pain by controlling cytokine regulation and immune cell migration (see Section 4.4.2.), and they also induce peripheral antinociception [19,89].

**Figure 3:**
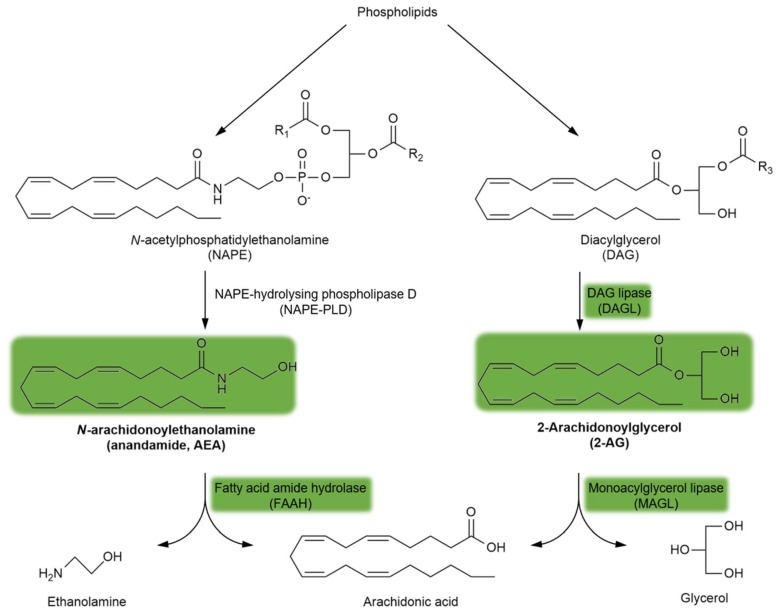
The synthesis and degradation of endocannabinoids. The green background indicates the metabolites and enzymes relevant to schizophrenia. Abbreviations of metabolites and enzymes frequently used in the text are also indicated.

Endogenous cannabinoid receptor (CBR) ligands are hydrophobic lipid-derived compounds, among which *N*-arachidonoylethanolamine (AEA) and 2-arachidonoyl glycerol (2-AG) have been most studied [90,91,92]. Their degradation is also important, with AEA being rapidly metabolized by the fatty acid amide hydrolase (FAAH) and 2-AG being hydrolyzed by the monoacylglycerol lipase (MAGL) enzyme (Figure 3). Blocking the FAAH enzyme has been considered a novel approach for the treatment of schizophrenia (see Section 5.4.). Furthermore, plant-derived phytocannabinoids, such as Δ^9^-tetrahydrocannabinol (Δ^9^THC), the major psychoactive component of cannabis, and the non-psychoactive cannabidiol (CBD), are also relevant to schizophrenia (see Section 3.2. and Section 5.4.). Importantly, the psychoactive effects of Δ^9^THC are mediated through the brain CB_1_R, the most abundant GPCR in the brain.

More than 30 years following the discovery and identification of CBRs, structurally diverse synthetic cannabinoids have been developed and synthesized to investigate their interaction with the ECS. Among these, the bicyclic CP 55940 and the aminoalkylindole WIN 55212-2 are potent CB_1_/CB_2_ agonists that represent important exogenous cannabinoids in the field of cannabinoid research. Later generations of synthetic cannabinoids, such as JWH-18, have been found in illicit herbal mixes (“Spice”) and classified as a Schedule I controlled substance [84,93] in the United States. Synthetic cannabinoids with high CB_1_R affinity and potency have been closely associated with the development of schizophrenia (see Section 3.2.).

### 3.2. The Cannabinoid Hypothesis of Schizophrenia

The cannabinoid hypothesis of schizophrenia has recently emerged, based on neuroimaging reports, postmortem studies, and clinical evidence. Within this hypothesis, we distinguish between the endogenous and exogenous cannabinoid hypotheses. The former is based on the fact that ECS deregulation has been observed among patients with schizophrenia [94]. Notably, alterations in CB_1_R availability, density, and/or mRNA expression and endocannabinoid levels have been reported in certain brain tissues and CSF of patients with schizophrenia [94,95,96,97]. On the other hand, the exogenous cannabinoid hypothesis refers to the association between environmental risk factors, such as frequent and/or early use of cannabis or synthetic cannabinoids, and the development of schizophrenia among vulnerable individuals, especially adolescents [10,98,99]. Δ^9^THC administration can induce positive and negative symptoms, as well as cognitive impairments, resembling those of schizophrenia among healthy individuals, while exacerbating symptoms among patients already diagnosed with schizophrenia [100,101,102]. Moreover, a study by Moore and coworkers showed that the risk of psychosis increases by approximately 40% among individuals who had previously used cannabis [103]. Although Δ^9^THC is mainly responsible for the connections between cannabis and schizophrenia, and while CBD can offset these associations (see Section 5.4), the cannabis plant itself contains a large variety of other phytocannabinoids, terpenes, and phenolic compounds, not to mention their metabolites [104]. This makes it challenging to accurately study the connection between cannabis consumption and the risk of schizophrenia development. 

## 4. Common Points and Potential Interactions between the Endocannabinoid System and Kynurenines Relevant to Schizophrenia

### 4.1. Overview

This main section will review the functions and mechanisms of kynurenines that overlap with the ECS and their potential interactions related to schizophrenia. Based on the literature, the following three main aspects that form the basis for known and potential interactions between kynurenines (mainly KYNA) and the ECS will be discussed in the subsequent sections: (1) glutamatergic, dopaminergic, and GABAergic neurotransmission, given that KYNA and CB_1_R regulate all three; (2) astrocytes, given their significance in KYNA production and CB_1_R function; and finally (3) inflammation associated with schizophrenia, given that both the KP and ECS play important roles in this mechanism. All three aspects will be discussed in separate sections while also underscoring the basics of astrocyte functioning and other related, yet undiscussed hypotheses of schizophrenia (dopaminergic, glutamatergic, and GABAergic neurotransmission and inflammation). Additionally, each section will be accompanied by tables summarizing the main studies related to the given section (see Tables 2–4).

### 4.2. Glutamatergic, Dopaminergic, and GABAergic Transmission Regulation by Kynurenines and the Endocannabinoid System in Schizophrenia

#### 4.2.1. The Basics of the Dopaminergic, Glutamatergic, and GABAergic Hypothesis of Schizophrenia

Dysregulation of brain neurotransmission, including dopaminergic, glutamatergic, and GABAergic systems, forms the basis for neurochemical theories on the etiology of schizophrenia [105]. Considering that all aforementioned transmitters are involved in the control of several cerebral processes, including locomotor functions, affect, motivation, and learning, abnormal activities therein have been thought to be associated with many schizophrenia symptoms [105,106,107,108].

While the mesolimbic dopaminergic pathway may play a role in the development of positive schizophrenia symptoms in the presence of excess dopamine and/or increased dopamine D_2_ receptor expression [109], negative symptoms and cognitive deficits are thought to be caused by low mesocortical dopamine levels and decreased dopamine D_1_ receptor density in the PFC [110]. However, clear limitations for this hypothesis exist, given that many aspects of schizophrenia cannot be explained based on dopaminergic dysfunction alone, and many patients remain persistently disabled despite treatment with various dopaminergic compounds.

Glutamatergic theories of schizophrenia have been based on the ability of NMDAR antagonists, such as phencyclidine (PCP) and ketamine, to induce schizophrenia-like symptoms and on disturbances of NMDAR-related gene expression and metabolic pathways accounting mainly for negative symptoms and some cognitive dysfunctions of the disorder [111,112,113]. Reduced NMDAR activity on inhibitory (GABAergic) neurons leads to disinhibition of glutamate neurons. Theoretically, such abnormally increased glutamatergic activity through AMPA and metabotropic glutamate (mGLUT) receptors causes overactivation of the mesolimbic and underactivation of the mesocortical dopaminergic pathways, leading to morphological and structural brain changes resulting in psychosis [113,114].

Postmortem studies have widely reported alterations in multiple GABA-related markers among patients with schizophrenia [115]. Dysfunction in the parvalbumin-containing subset of cortical inhibitory neurons together with both pre- and postsynaptic components of GABAergic neurotransmission could also play an important role in the clinical features of schizophrenia [108,116]. One of the most consistent postmortem findings in schizophrenia is reduced glutamic acid decarboxylase 67 (GAD 67) mRNA expression and consequent attenuation of inhibitory GABAergic neurotransmission across multiple brain areas affected by schizophrenia [108,117]. These abnormalities could create disturbances mainly related to emotional functioning and cognitive control. Additionally, one clinical study reported lower GABA concentrations in CSF samples from patients with first-episode psychosis compared with those from healthy volunteers, which were associated with total and general Positive and Negative Syndrome Scale scores, illness severity, and poor performance in a test of attention [118]. However, neuroimaging studies measuring in vivo GABA have revealed no consistent alterations in schizophrenia that might be hypothesized from animal models and postmortem data [119]. The absence of large, detectable differences in GABA concentrations could reflect normalization via compensatory upstream mechanisms that tend to increase the synaptic activity of GABA [115], which include the reduction in GABA transporter 1 mRNA expression on presynaptic neurons (responsible for GABA reuptake) and upregulation of GABA_A_ receptors in postsynaptic pyramidal neurons [108,120].

KYNA and cannabinoids have been known to modulate the abovementioned neurotransmissions, which will be discussed below and summarized in Table 2.

#### 4.2.2. KYNA and Dopaminergic/Glutamatergic/GABAergic Interactions in Schizophrenia

Preclinical studies have provided ample evidence to suggest that KYNA has an inverse bidirectional relationship with several neurotransmitters, including glutamate, dopamine, and GABA, which could contribute to all symptom domains of schizophrenia [7,8]. Accordingly, though KYNA is generally considered to be protective against QUIN-induced excitotoxicity, its abnormal accumulation beyond physiological concentrations may cause NMDAR hypofunction on cortical GABA interneurons. This may lead to reductions in GABAergic neurotransmission and disinhibition of cortical glutamatergic projections [128], as well as an excitatory effect on ventral tegmental area (VTA) dopamine firing induced by the blockade of the NMDAR glycine site. Meanwhile, electrophysiological studies have shown that KYNA appears to have an opposite action on dopamine neurotransmission via α7nAChR antagonism, consequently reducing dopamine release and promoting cognitive impairments [121].

#### 4.2.3. The Endocannabinoid System and Dopaminergic/Glutamatergic/GABAergic Interactions in Schizophrenia

Given that CB_1_Rs inhibit the release of several neurotransmitters, including dopamine, GABA, serotonin, glutamate, noradrenaline, and acetylcholine, the ECS may be considered a key neuromodulatory pathway relevant in the etiology of multiple mental disorders [10]. Increasing evidence has suggested complex functional interactions between these neurotransmitter systems at the anatomical and pharmacological levels. Generally, endocannabinoids are released on demand by the postsynaptic neurons and travel retrogradely across the synapse, binding to and activating CB_1_Rs located on the presynaptic terminals [125]. Such activation results in the short- or long-term decrease in neurotransmitter release [126].

VTA dopaminergic cells can be considered a hub between brain regions processing sensory and cognitive information that use the endocannabinoid lipid molecules as metabolic and homeostatic signal detectors, influencing cell function [125]. The effects of cannabinoids/endocannabinoids on dopamine transmission and dopamine-related behaviors are generally indirect and exerted through decreased neurotransmission [94]. Thus, cannabinoid agonists reduce glutamate release from hippocampal neurons [129], which results in a net increase in cortical pyramidal neuron excitability via the activation of CB_1_Rs located on inhibitory GABAergic cells [127]. However, Steffens et al. had demonstrated that the existence of CB_1_Rs in human neocortical dopamine terminals also directly affects cortical dopamine input [130]. All these mechanisms likely contribute to cannabinoid-induced learning and memory impairments. Furthermore, certain endocannabinoids (e.g., *N*-arachidonoyl dopamine and AEA) may directly activate transient receptor potential vanilloid 1 channel (TRPV_1_) receptors [125,131], thereby allowing direct facilitatory regulation of dopamine function (e.g., at the nucleus accumbens) that influences the motivated behavior and reward process [9].

### 4.3. Astrocytes as a Potential Stage for the Endocannabinoid System and Kynurenine Interaction in Schizophrenia

#### 4.3.1. Overview of Astrocytes and Their Role in Schizophrenia

For many years, astrocytes were believed to be passive brain elements that maintain structural and metabolic support for neurons [12]. However, recent studies have clearly demonstrated that astrocytes are vital functional components of synapses, forming the so-called tetrapartite synapse, including pre- and postsynaptic elements, other distinct glia cells aside from astrocytes (e.g., NG2 or microglia), and the extracellular matrix [132,133,134]. In the tetrapartite synapse, astrocytes together with the extracellular matrix create a synaptic cradle providing the basis for essential processes contributing to neuroplasticity, such as synaptogenesis and synaptic maturation, isolation, and maintenance [135]. Accordingly, one recent review reported that each element of the tetrapartite synapse is disrupted in schizophrenia [136]. CB_1_Rs and certain enzymes of the KP in astrocytes have been strongly associated with schizophrenia and will be reviewed in this section, together with KYNA and its receptor targets in astrocytes. Moreover, Table 3 summarizes the participating members for kynurenines and associated elements and the ECS, as well as their common points, in astrocytes involved in schizophrenia.

#### 4.3.2. CB_1_Rs, KYNA Production, and Target Receptors of KYNA in Astrocytes

CB_1_Rs located on astrocytes are particularly interesting given their very low expression levels therein [141,143,144], which is in contrast to their significance in terms of synaptic transmission, long-term synaptic plasticity, and thus working memory [145,146,147]. Another interesting aspect of astrocyte-derived CB_1_Rs is their coupling to G_q/11_ type G-proteins, which activates phospholipase C and produces inositol triphosphate [147]. This differs from the more widespread G_i/o_ type coupling, which inhibits adenylate cyclase and cAMP production [78]. Additionally, 2-AG and AEA endocannabinoids are also produced in astrocytes [122,123]. In fact, CB_1_Rs and the 2-AG synthesizing enzyme, diacylglycerol lipase (DAGL; Figure 3), are co-expressed in close vicinity, although this was demonstrated in spinal astrocytes from rats [139]. Moreover, MAGL, the enzyme responsible for 2-AG degradation (Figure 3), is also expressed in astrocytes [140].

Astrocytes are key players in the KP given that KYNA synthesis (i.e., the irreversible transamination of l-KYN to KYNA via KAT enzymes) takes place almost exclusively in such cells throughout the mammalian brain [35]. Among the KAT enzymes, the type II enzyme is responsible for approximately 75% of KYNA production in the mammalian brain under normal conditions [137] and can be found mainly in astrocytes [148], with l-KYN being its only endogenous substrate [33,149]. Additionally, KYNA-producing astrocytes are positioned close to the capillary walls and pericytes of the blood–brain barrier, which allows these glia cells to effectively accumulate l-KYN from the circulation and quickly respond to fluctuations in peripheral KYN concentrations [150,151,152,153].

α7nAChRs, which are functionally expressed in astrocytes, have been implicated in memory functions and neuroprotection [138,154]. Given the low abundance of NMDARs, demonstrating their presence and functionality in astrocytes has remained challenging. Nevertheless, studies have shown that astrocytic NMDARs are constructed from the same set of seven subunits, albeit differently configured and assembled compared with neuronal NMDARs [155]. It is now clear that astrocytic NMDAR activation generates intracellular calcium signaling, which—at least in hippocampal astrocytes—has been suggested to enhance the release of inhibitory gliotransmitters (e.g., ATP or endocannabinoids), eventually modulating presynaptic strength [156]. However, further studies are needed to explore the effect of astrocytic NMDARs on neurotransmission modulation. To date, functionally active GPR35 receptors have only been demonstrated in cultured astrocytes, in which the activation of such receptors via KYNA reduces forskolin-induced cAMP production and ATP-induced calcium transients [48].

#### 4.3.3. The Role of Astrocytic CB_1_Rs, α7nAChRs, and KYNA in Glutamate Neurotransmission and Its Significance in Schizophrenia

Astrocytes play a significant role in glutamate biosynthesis, glutamate–glutamine cycle, glutamate uptake and release, and d-serine biosynthesis and release, all of which are known to be dysregulated in schizophrenia [157]. The role of CB_1_Rs and α7nAChRs in astrocytic glutamate neurotransmission has been studied in detail. Accordingly, activating the aforementioned receptors stimulates glutamate release, whereas blocking them inhibits this process [145,158], thereby modulating neuronal excitability. In fact, studies have demonstrated that astrocyte-derived KYNA reduces glutamate release in the PFC through α7nAChR. A recent study by Secci and coworkers revealed that CB_1_R and α7nAChR mRNA co-localize on rat cortical astrocytes in the medial PFC [142] and are involved in the THC-induced increase in glutamate release within the same region given that it was inhibited by both rimonabant and KYNA [142]. Evidence has shown that cannabis use can reduce the negative symptoms of schizophrenia [159,160], which Secci and coworkers found to be in agreement with their results. In other words, excessive KYNA levels in the medial PFC associated with schizophrenia reduce astrocytic glutamate release through the inhibition of α7nAChR, resulting in glutamate and NMDAR hypofunction in the medial PFC, which is also attributed to the disorder. Thus, cannabis can attenuate astrocytic-derived glutamate hypofunction and potentially improve the symptoms associated with schizophrenia. Additionally, astrocytic CB_1_Rs and KYNA via α7nAChRs may secondarily modulate dopamine release and the reinforcing properties of THC [161,162,163,164,165].

### 4.4. The Involvement of Kynurenines and the Endocannabinoid System in the Inflammatory Component of Schizophrenia

#### 4.4.1. The Inflammatory Hypothesis of Schizophrenia

Numerous genetic, epidemiological, and clinical evidences have suggested that inflammatory pathways are disrupted in schizophrenia. Moreover, several studies have demonstrated that individuals with infection or autoimmune diseases are more susceptible to schizophrenia [166,167,168,169,170]. The inflammations associated with schizophrenia, as will be discussed in the following section, are related to both the CNS and peripheral organs, especially GI inflammation. Several studies have demonstrated that both the ECS and kynurenines, as well as their related enzymes and receptors, are involved in inflammation and immune regulation [13,19,111,171]. Although no reported evidence has yet suggested crosstalk between these two systems in the inflammatory hypothesis of schizophrenia, many common points indicate its possibility, including inflammatory cytokine regulation, microglial activation, oxidative stress, GI inflammation, and related microbiome regulation, which will be explored in the following sections. Participating members and common points in the described mechanisms are summarized in Table 4.

#### 4.4.2. Neuroinflammation, Cytokines, and Microglia Activation

A substantial amount of data has shown that acute and chronic CNS inflammation, which can be induced by infectious agents, environmental toxins, factors, neural lesions, or genetic defects, is associated with schizophrenia [192,193]. Several inflammatory degradation products, among which inflammatory cytokines are the most significant [192], have been observed in brain tissues and the CSF of approximately 50% of patients with schizophrenia [194,195]. Inflammatory cytokines are important mediators in the communication between the CNS and immune system, with previous studies thoroughly demonstrating their imbalance in schizophrenia [196,197]. Considering that microglial dysfunction is also a significant factor in the development of inflammation and schizophrenia, the microglial hypothesis has been another suggested mechanism contributing to the pathology of the disorder [193,198,199,200,201,202]. Microglia are the main components of the immune system of the CNS. Accordingly, systemic inflammation activates microglia, which in turn produce and release proinflammatory cytokines and reactive oxygen species (ROS), increasing blood–brain barrier permeability [203]. This allows inorganic and organic toxins to more easily enter the CNS, contributing to neurological diseases, such as schizophrenia [204]. Microglial overactivation leads to microglial sensitization or priming, wherein microglia will subsequently induce an exaggerated immune response to a weak stimulus in the form of higher levels of cytokine production/release and microglial proliferation [205,206], which can influence the development of schizophrenia [192].

Studies have shown a link among inflammation, Trp metabolism/KP, and schizophrenia [111]. Proinflammatory cytokines, such as interferon-γ (IFN-γ), interleukin 1, and tumor necrosis factor alpha (TNFα), are able to shift Trp metabolism to l-KYN by increasing IDO enzyme activity [173,174,207,208]. Accordingly, IDO1 expression and enzymatic activity have been demonstrated to be upregulated in response to infection, resulting in the accumulation of l-KYN and 3-HK, which possess antimicrobial activity [172]. Interestingly, no pathogen has thus far shown sensitivity to KYNA [172], which has been demonstrated to have anti-inflammatory and immunosuppressive properties [15]. These properties are mainly mediated through GPR35 and AHR receptors [15]. Multiple studies have found an association between *Toxoplasma gondii*, an obligate intracellular protozoan parasite that causes the infectious disease toxoplasmosis, and schizophrenia [209,210,211,212,213]. This parasite has been suggested to increase IFN-γ production, which activates IDO in microglia leading to Trp degradation and L-KYN elevation [214,215]. Consequently, the concentration of other kynurenines increases dramatically, including KYNA in astrocytes, which were at the peak level 28 days post-infection and continued elevating after 56 days [213]. This persistent brain KYNA elevation may contribute to the cognitive impairment observed in schizophrenia [212]. The KAT enzyme, which seems to be cell-type specific, has also been involved in inflammatory regulation. Reports have shown that IFN-γ alone or in combination with TNF reduced KAT II, III, and IV mRNA expression in human dermal fibroblast cells [175]. Interestingly, the same study revealed that KYNA levels were increased in the presence of IFN-γ. In fetal astrocytes, IFN-γ increased the level KAT I and II transcripts [35], whereas lipopolysaccharide treatment also increased KAT I but reduced KAT II mRNA expression in the hippocampus [216].

The ECS plays a key role in immunomodulation. Accordingly, both exogenous cannabinoids and endocannabinoids suppress the production and release of proinflammatory cytokines in both peripheral organs and the CNS through CB_2_Rs [19,179]. Another study demonstrated higher CBR availability on innate immune cells and a simpler correlation network between cytokines and CBR expression among patients with schizophrenia than among controls [217]. Circulating endocannabinoid levels have been known to increase several fold during systemic inflammation [176]. This seems to be supported by the finding of increased AEA levels in the CSF of patients with schizophrenia, although it is negatively correlated with psychotic symptoms in the disorder [96]. Interestingly, studies have reported a positive correlation between 2-AG levels and proinflammatory cytokine interleukin 6 concentrations [177,178]. Patients with borderline personality disorder share most of the positive symptoms with those with schizophrenia and exhibit significantly higher circulating 2-AG and AEA levels compared with controls [218]. As discussed in the previous sections, cannabis consumption is a potential risk for the development of schizophrenia in vulnerable individuals, such as adolescents. Additionally, immunomodulation can be one of the causal background mechanisms of cannabis. Δ^9^THC has also been shown to reduce cytokine production and secretion in most immune cells of the CNS. Cytokines play a significant role in neurodevelopment and modulation of neurotransmitter and neuropeptide systems, including the monoamine system [219], which might explain why adolescence is the most susceptible period for cannabis smoking. Exogenous cannabinoids can also modify microglia functioning and thus alter neurotransmission release and neuron architecture [198,220,221,222]. Additionally, studies have reported that both GPR35—of which the KYNA is an endogenous ligand—and CB_2_R are expressed on leukocytes and involved in leukocyte recruitment, which can be induced by KYNA in the case of GPR35 [223,224,225,226,227,228,229]. In fact, GPR35 and CB_2_R (and CB_1_R) have similar structures and receptor signaling pathways [49,78], with studies suggesting a linkage between GPR35 and cannabinoid receptors through the interconversion of their endogenous ligands, 2-acyl lysophosphatidic acid and 2-AG [189]. Thus, the aforementioned data may indicate a potential interaction between GPR35-mediated KYNA signaling and CB_2_R in inflammatory processes associated with schizophrenia.

#### 4.4.3. ROS and Oxidative Stress

ROS, such as superoxide or hydroxyl radicals, are byproducts of several enzymatic reactions related to basic metabolic functions occurring in certain cell compartments, such as mitochondria, peroxisome, endoplasmic reticulum, cell membrane, or cytoplasm [230]. Oxidative stress refers to the imbalance between ROS and the class of protective reduction–oxidation enzymes that detoxify ROS, such as catalase, superoxide dismutases, and enzymes of the glutathione system (e.g., glutathione peroxidases) [231]. Inflammatory processes are tightly associated with oxidative stress and ROS production given that the immune system starts to intensely produce ROS in response to infection, which partly elicits inflammation via immune cell and microglial cytokine production [231]. Inflammatory cytokines, such as TNFα and interleukins 1 and 10, or other inflammation-inducing signals, such as lipopolysaccharide, thrombin, or oscillatory shear stress, affect ROS production. Increased ROS levels can activate nuclear factor κ-light-chain enhancer of activated B cells (NF-κB), which then induces downstream mechanisms, such as antioxidant and inflammatory gene transcription or proteasome and inflammasome activation [230]. Considerable data have demonstrated increased oxidative stress in patients with schizophrenia, indicated by increased DNA, lipid, and protein oxidation and increased levels of total ROS accompanied by reduced gene levels of antioxidant enzymes [231,232]. Additionally, patients with schizophrenia exhibit mitochondrial dysfunction, which induces oxidative stress and inflammatory processes [233]. As such, studies have suggested that oxidative stress ties together certain risk factors of schizophrenia, such as aberrant neuronal migration, synapse formation, neurotransmission, or neuroinflammation [231].

The KP plays a significant role in maintaining antioxidant balance in the brain. Persistent oxidative stress via an imbalanced KP may lead to disrupted glutamatergic and dopaminergic neurotransmission and altered brain functioning (see Section 4.2.) [192,196]. Certain metabolites of the KP (see Section 2.1.1.) can generate oxidative stress and ROS, such as 3-HK, 3-HA, or QUIN [16,180,181,182,234,235,236], with QUIN also being able to induce lipid peroxidation and mitochondrial dysfunction [16,235,237,238,239,240]. On the other hand, KYNA behaves as an endogenous antioxidant by scavenging free radicals and inhibiting oxidative stress [182,236,238]. Additionally, a quantitative analysis of schizophrenia-associated serum metabolites revealed low levels of the antioxidant glutathione accompanied by increased levels of Trp and kynurenine [241].

The link between the ECS and redox homeostasis has now become evident given the numerous studies revealing the neuroprotective effects of cannabinoid ligands [183,242,243]. Furthermore, endocannabinoids are significantly involved in cell ROS production given that they control mitochondria-derived ROS generation [20] notably by altering the expression and/or activity of mitochondrial electron-transport chain components and/or by promoting changes in mitochondrial membrane potential via the CB_1_R [244]. The ECS and related endocannabinoids can also regulate oxidative stress and lipid peroxidation either through both CBRs or by scavenging free radicals [184,245]. Interestingly, CB_1_R and CB_2_R are distinctly involved in oxidative stress regulation, depending on the cell and injury type and disease progression [245]. Accordingly, CB_1_R activation enhances redox imbalance, whereas CB_2_R activation lowers ROS production [20,21,184]. 

#### 4.4.4. Gastrointestinal Inflammation and Gut Microbiome

Considering that the GI tract is our body’s largest immune organ and is connected bidirectionally to the brain through multiple neuronal pathways, disruption in GI function can affect the brain and has been linked to the development of schizophrenia [246]. Given that the gut–immune barrier and blood–brain barrier are functionally and structurally similar [247], the hypothesis is that toxic and bioactive compounds penetrate through the epithelial and endothelial barriers of both the GI tract and CNS, thereby inducing an immune response [246]. Schizophrenia has also been associated with GI inflammatory comorbidities, such as irritable bowel syndrome (IBS) and inflammatory bowel diseases (IBD) [248,249]. The involvement of the gut microbiome in the inflammatory component of schizophrenia has also been an emerging field. Accordingly, a bidirectional relationship has been suggested, given that changes in the microbial flora of the gut might lead to schizophrenia or other neuropsychiatric disorders [250,251], while the brain can also alter the microbial habitat and composition in the GI [252]. Studies have reported abnormal microbiome function, composition, and amount in the oropharynx and feces of drug-naive patients with schizophrenia [253,254,255,256,257]. Interestingly, risperidone—the most common medication for schizophrenia—has been shown to alter fecal bacterial composition [258].

KYNA has been extensively studied in the GI system. Interestingly, KYNA content gradually increases along the GI tract, with the distal-most portion having the highest content [14]. Considering the positive correlation observed between KYNA content and microflora concentration in the small intestine [186], the gut flora has been suggested to produce the common pool of intestinal KYNA [14]. Notably, certain food and herbs, such as honey, broccoli, or basil, also contain KYNA in micromolar concentrations [259,260]. Additionally, KYNA may possess both negative and positive effects in bowel diseases [14]. Accordingly, serum KYNA levels are increased in IBS most probably as a compensatory mechanism [186] but are reduced in IBD [186]. Moreover, studies have shown that KYNA stimulates bacterial growth in the GI system at low and medium concentrations [187] but displays antimicrobial activity at high concentrations [261]. The GI-related effects of KYNA are mediated through GPR35 [14], which is highly expressed in the GI tract [49,190] and has been associated with IBD [191].

Endocannabinoids have been known to communicate with the gut microbiome [185] while also playing an important role in regulating intestinal microbial product entry into the bloodstream and thus in the development of metabolic diseases [18,19]. Additionally, multiple studies have highlighted the therapeutic relevance of the ECS in IBD and IBS [18,188]. Cannabinoid receptors are abundantly expressed in different areas/cells of the GI system, such as on enteric nerves, enteroendocrine cells, immune cells, and enterocytes [19]. Similar to GPR35, cannabinoid receptors have also been implicated in IBD [262]. Thus, considering the previously discussed overlapping functional and structural properties of cannabinoid and GPR35 receptors, their high expression levels in the GI system, and their common involvement in IBD, another potential area for their interaction within the inflammatory component of schizophrenia can be surmised.

## 5. Therapeutic Potentials

### 5.1. Overview

This section will highlight the therapeutic potentials of the KP and ECS in the treatment of schizophrenia. Numerous studies have investigated KAT II inhibitors and CBD, which will be reviewed here (also see Table 1 and Table 5). The most appealing approach would be to combine both types of compounds to achieve a synergistic and more efficacious therapeutic effect. Additionally, these alternative therapeutic targets might improve the main limitations of currently available medications, namely, their poor effect on negative symptoms and cognitive impairment, as mentioned in the introduction. A separate section will discuss the currently available dopaminergic antipsychotic medications and clinical studies of non-dopaminergic agents in order to assess the potential of KAT II inhibitors and CBD.

### 5.2. Currently Available Medications

The goals in treating schizophrenia include targeting symptoms, preventing relapse, and increasing adaptive functioning through both pharmacological and non-pharmacological (such as psychotherapy) treatments whereby the patient can be integrated back into the community [105,276].

Antipsychotic drugs (APDs), which have been primarily used to manage psychosis (including hallucinations, delusions, disordered thought, or paranoia), have been the mainstay of pharmacological treatment protocols in schizophrenia as recommended by the National Institute of Health and Care Excellence, World Health Organization, and the American Psychiatric Association [277,278,279]. All clinically approved and currently used APDs have nanomolar affinity for the dopamine D_2_ receptor and fully or partially block the actions of dopamine in the mesolimbic pathway [280].

Over the past 50 years, numerous first-, second-, and third-generation antipsychotics have been developed, while dramatic growth in the research of pharmacological schizophrenia treatment has advanced our understanding of the neurobiology and neuropharmacology of the illness [279,281,282]. First discovered in the 1950s, first-generation antipsychotics (e.g., chlorpromazine, haloperidol, and fluphenazine), known as typical APDs, not only have antipsychotic effects but also extrapyramidal side effects, and cause hyperprolactinemia in association with their full D_2_ receptor antagonism in the CNS. First-generation antipsychotics also possess high affinity for muscarinic M_1_ ACh, histaminergic H_1_, and α_1_ norepinephrine receptors, which can result in partially distinctive side-effect profiles (e.g., cognitive deficits and sedation) [283].

Since the 1990s, newer drug compounds (clozapine, risperidone, olanzapine, quetiapine, etc.) that blocked both dopamine and serotonin receptors were met with great expectations [284,285] and were found to be effective in alleviating both positive and negative symptoms [105]. Although the introduction of second-generation antipsychotics had become a cornerstone in the treatment of schizophrenia, several unmet treatment needs in the field still existed. While newer antipsychotics produced fewer motor side effects, safety and tolerability concerns regarding metabolic side effects, such as obesity, dyslipidemia, and type 2 diabetes, have emerged [286].

Third-generation antipsychotics (e.g., aripiprazole and cariprazine), which are partial D_2_ agonists, represent another pharmacologically different strategy in the attempt to normalize dopaminergic imbalance in schizophrenia. Compared with full agonists, these agents have lower intrinsic activity at D_2_ receptors, allowing them to act as either functional agonists or antagonists, thereby inhibiting endogenous dopamine activity in the mesolimbic and activating the mesocortical pathways [287,288]. In addition, such an agent should ideally maintain dopaminergic tone in the nigrostriatal and tuberoinfundibular pathways, thereby preventing extrapyramidal symptoms and hyperprolactinemia. Additionally, they usually have partial agonist properties at dopamine D_3_, D_4_, 5-hydroxytriptamin (5-HT)_1A_, 5-HT_2C_, and, to a much lesser extent, 5-HT_2A_ receptors [289,290].

Considering that nearly 30% of patients do not respond to dopaminergic antipsychotics, treatment resistance in schizophrenia and the need for decreasing serious adverse effects (extrapyramidal and metabolic) associated with their long-term use have remained as major issues in psychiatry [291]. Findings regarding the inefficiency and safety profile of APDs have prompted the discovery of promising new targets for the development of non-dopaminergic drugs based on the glutamatergic and GABAergic hypothesis of schizophrenia that may replace currently used treatments. These will be reviewed briefly in the following section.

#### Non-Dopaminergic Agents in Clinical Studies Based on the Glutamatergic and GABAergic Hypothesis of Schizophrenia

Several approaches have been used in restoring NMDAR hypofunction [114]. While classical NMDAR agonists have not been useful given that their excessive stimulation results in excitotoxicity and neuron damage, the modulatory mechanisms of NMDAR functioning have been considered as more promising targets [113,292,293]. Clinical trial results regarding NMDAR-enhancing small molecules as an adjunct to dopaminergic drugs, such as glycine and d-serine (endogenous full agonists of the NMDAR glycine site) and D-cycloserine (a partial NMDAR agonist), have been inconsistent [294,295,296,297,298,299,300]. Memantine, a drug that acts as a weak nonselective NMDA receptor antagonist, had been associated with significant attenuation of positive, negative, and cognitive symptoms when used as an add-on treatment to clozapine or olanzapine [301,302]. Positive allosteric modulators of AMPA-type glutamate receptors, such as ampakines, and glycine transporter blockers, such as *N*-methylglycine (sarcosine), have also been considered as promising therapeutic agents used in adjunct to already available dopaminergic antipsychotics [303,304,305,306]. Preclinical studies have suggested that compounds targeting metabotropic glutamate receptors, specifically subtype-selective allosteric modulators, may also be used as an alternative to current treatments [114,307].

One pilot study involving a 4-week treatment with MK-0777, a partial GABA(A) receptor agonist, revealed progress in cognitive performance among patients with chronic schizophrenia, providing support for the beneficial effect of enhanced GABA activity in prefrontal functioning [308]. However, a later clinical study involving 60 patients with schizophrenia showed little benefit [308]. Thus, more potent partial agonists with greater intrinsic activity at the GABA(A) α2 site might be needed for cognitive enhancement in schizophrenia.

In conclusion, the abovementioned non-dopaminergic drugs have little to no effect when used by themselves, but may improve the negative symptoms and cognitive impairments when used as adjunct treatment to dopaminergic drugs without significant safety concerns. Based on these clinical findings, compounds targeting the KP and ECS could be a compelling alternative approach toward satisfying the unmet clinical needs of patients with schizophrenia.

### 5.3. Targeting the KP

Pharmacological manipulation of the KP for the treatment of schizophrenia is a complex approach as described by Müller and colleagues [26]. Although increased brain KYNA levels have now been considered as an important factor contributing to the complex symptoms of the disorder, reducing KYNA levels could impair its neuroprotective effect against, for example, QUIN-induced excitotoxicity [309]. Nevertheless, while many studies have dealt with this subject, KAT enzyme targeting has been the most intensely studied therapeutic approach against schizophrenia.

As discussed in Section 2.1.1., KATs are responsible for the irreversible transamination of l-KYN to KYNA [33], mainly in astrocytes. Thus, inhibiting KAT enzyme activity can be considered as a logical approach for reducing increased brain KYNA levels associated with schizophrenia. This approach would be less likely to interfere with other parts of the KP [310]. As described in Section 4.3.2., KAT II has the greatest potency for therapeutic targeting among the four KAT enzymes owing to its substrate specificity and its role in the production of most of the KYNA in the brain. Studies have shown that reducing brain KYNA concentrations significantly improves cognitive functions through selective inhibition of the KAT II enzyme [70,311]. While multiple KAT II inhibitors have been developed to date, earlier designs, such as (S)-ESBA and BFF-122, were able to increase extracellular levels of dopamine, acetylcholine, and glutamate and improve memory functions in rats with schizophrenia-like symptoms [63,312,313,314,315]. However, due to poor blood–brain barrier penetration, these earlier compounds required intracerebral administration to achieve central effects. Such compounds were followed by systematically active, brain-penetrant KAT II inhibitors, such as PF-04859989 [316] and BFF-816 [311]. Accordingly, PF-04859989 irreversibly inhibited both rat and human KAT II, acutely inhibited amphetamine- and ketamine-induced disruption of auditory gating, and improved performance in a sustained attention task. Moreover, it prevented ketamine-induced disruption of performance in a working and spatial memory task in rodents and nonhuman primates, respectively [70]. These behavioral experiments were confirmed by electrophysiological studies, wherein PF-04859989 reduced the activity of midbrain dopamine neurons and nicotine-evoked glutamatergic activity in the rat cortex [317,318]. Other compounds have been developed to improve the pharmacological properties of PF-04859989 [22,319]. In contrast to PF-04859989, BFF-816 reversibly inhibited KAT II, improved performance in spatial and contextual memory, attenuated evoked glutamate release in rat PFC, and decreased hippocampus-dependent memory deficits in adult rats prenatally treated with kynurenine [54,311,320]. Additionally, previous studies have reviewed several other design approaches for KAT II inhibition [22,23,24,25,27].

Apart from KAT II, limited studies have examined other KP enzymes as a therapeutic target for schizophrenia. One recent study describing an animal model of schizophrenia induced by ketamine revealed that IDO, TDO, and KMO inhibition improved behavioral changes, prevented lipid peroxidation and protein damage, and protected against antioxidant enzymes in rats [321]. IDO, in particular, gained more attention due to its previously discussed role in inflammation associated with the disease [7,26,263].

### 5.4. Targeting the Endocannabinoid System

A considerable amount of data has suggested a connection between excess Δ^9^THC and synthetic cannabinoid consumption and the development of schizophrenia. However, recent evidence has also shown the positive effects of cannabinoid compounds in patients with schizophrenia. For instance, one study showed that dronabinol, the synthetic variant of Δ^9^THC, reduced core psychotic symptoms in three out of six treatment-refractory patients with severe chronic schizophrenia, who had a self-reported history of improvement with marijuana abuse [272].

Among cannabinoid compounds, CBD appears to be the most promising for the treatment of schizophrenia. CBD, the other main component of cannabis, does not possess psychoactive properties as mentioned previously. In fact, some of the effects of CBD on brain function and psychiatric symptoms contrast those of Δ^9^THC [322]. In contrast, a recent study reported that CBD does not attenuate Δ^9^THC-induced acute psychosis and memory impairments [102]. The precise mode of action of CBD has yet to be fully understood given that it has mixed pharmacological properties, including a week antagonistic binding toward CBRs, inhibition of FAAH activity, and stimulation of TRPV1, the 5-HT1A receptor, and the D_2_ dopamine receptor [323,324]. Moreover, Bih and coworkers revealed that numerous additional receptors, transporters, ion channels, and enzymes that could serve as molecular targets for CBD are involved in neurological disorders [325]. According to preclinical studies, CBD reduced amphetamine-induced effects on prepulse inhibition and hyperlocomotion induced by other psychotomimetic drugs [265,326]. Human studies have shown that CBD improved both positive and negative symptoms of schizophrenia [264,266,327,328,329]. Accordingly, studies that showed negative results provided either a single dose or monotherapy of CBD [330,331] or included patients with chronic schizophrenia who received multiple antipsychotic medications [102]. Furthermore, compared with the conventional antipsychotic amisulpride, CBD reduced schizophrenia symptoms but with significantly less side effects [266]. The same study also showed that CBD increased serum AEA levels, which was associated with symptom improvement. This can be explained by the ability of CBD to block FAAH activity, although other mechanisms have been proposed for its antipsychotic effects (e.g., via the already mentioned D_2_, 5-HT1A and TRPV1 receptors) [325,332].

Studies have shown that AEA levels are inversely correlated with the severity of negative schizophrenia symptoms [96], which leads to the assumption that high AEA levels might be advantageous in schizophrenia. Thus, selective FAAH inhibition has also been extensively studied apart from CBD. Accordingly, blocking AEA degradation improved both PCP- and amphetamine-induced positive and negative symptoms in animals [267,268]. URB597, a selective FAAH inhibitor, reversed PCP-induced social withdrawal effects and associated changes in c-Fos activation/inactivation observed in distinct neuroanatomical locations related to the social interaction neurocircuitry [333]. Selective FAAH inhibition also alleviated the hyperdopaminergic phenotype of adult rats [270]. However, a novel schizophrenia rat model showed that during adolescence, URB597 treatment—which is similar to exogenous cannabinoid treatment—increased the proportion of susceptible rats developing increased dopamine neuron activity [269]. Unlike exogenous cannabinoid, however, URB597 did not alter the behavioral response to amphetamine. Finally, a study on mouse hippocampal neuronal cell lines revealed that AEA was a potential candidate for the treatment of oxidative stress-related neurological disorders. The same study showed that during H_2_O_2_-induced redox imbalance, AEA increased intracellular levels of superoxide dismutase and glutathione via CB_1_R, thereby protecting the cells from oxidative stress [271].

The higher CB_1_R density and/or endocannabinoid levels in certain cortical and subcortical (limbic) structures in patients with schizophrenia might also be associated with dopaminergic neuron hyperactivity (positive symptoms) and glutamate neuron hypoactivity (negative symptoms) [9]. Preclinical studies have revealed that the antipsychotic potential of the CB_1_R antagonist rimonabant was related to alterations in dopamine and glutamate transmissions in cortical structures [273,274]. Moreover, a 16-week double-blind, placebo-controlled, randomized clinical trial showed that rimonabant did not improve global cognitive functioning, but did improve a specific learning deficit based on the response to positive feedback [275]. Furthermore, one study showed that the rimonabant group exhibited a significantly better total Brief Psychiatric Rating Scale score and anxiety/depression and hostility factors compared with placebo-treated patients with schizophrenia [275]. However, another placebo-controlled clinical trial showed no improvements [334].

## 6. Summary and Conclusions

Schizophrenia has many aspects in which both kynurenines and the ECS are involved. Although both have already been separately reviewed in detail, their overlapping functions, mechanisms, and potential interaction in schizophrenia have yet to be elucidated. Therefore, the present review aimed to highlight such aspects. Accordingly, the most well-known overlapping areas include dopaminergic, glutamatergic, and GABAergic transmission regulation via cannabinoids and KYNA. Moreover, the most possible receptor mediator for KYNA in this mechanism is the astrocytic α7nAChR given that NMDAR inhibition by KYNA does not seem to influence glutamate release [61]. Inflammatory mechanisms contributing to the development of schizophrenia are complex and widespread and need to be studied more thoroughly. The overlapping structural, pharmacological, and anatomical properties between GPR35 and CBRs are also promising candidates for regulating the common aspects of inflammation associated with schizophrenia.

Though the treatment of schizophrenia still remains challenging, a better understanding of the possible connections between kynurenines and the ECS could introduce novel therapeutic compounds and targets for treatment. Such compounds could also compensate for limitations of currently available medications. While KAT II inhibitors and CBD are promising, it will be interesting to determine whether co-administration would yield a synergistic effect. Nonetheless, additional studies are needed to adequately explore the interaction between kynurenines and the ECS and to better understand their separate functioning.

Finally, parallel alterations in kynurenines/the KP and the ECS are present not only in schizophrenia but also in other neurological disorders, such as Alzheimer’s disease [38,335,336,337]. Thus, studying the interaction between kynurenines and associated elements and the ECS might also help us further understand mechanisms and disorders apart from schizophrenia.

## Figures and Tables

**Table 1:** The main studies reviewing aspects of schizophrenia that are shared by kynurenines and the endocannabinoid system (ECS). Reviews discussing the main therapeutic targets for kynurenines and the ECS relevant to schizophrenia are also indicated separately.

Common Points and Therapeutics	Kynurenines	ECS
Glutamatergic, dopaminergic, and GABAergic systems	[7,8]	[9,10]
Astrocytes	[11]	[12]
Inflammation	[13,14,15,16,17]	[18,19,20,21]
Therapeutics	[7,22,23,24,25,26,27]	[28,29,30]

**Table 2:** Kynurenines and associated elements (enzymes, receptors) and members of the ECS participating in glutamatergic, dopaminergic, and GABAergic neurotransmission associated with schizophrenia.

Members	References
***Kynurenines and associated elements***	
KYNA	[7,8]
α7nAChR	[121]
***ECS***	
AEA, 2-AG	[122,123,124]
CB_1_R	[10,125,126,127]

**Table 3:** Kynurenines and associated elements (enzymes, receptors) and members of the ECS present in astrocytes and involved in schizophrenia. The table also highlights the common points between the two systems.

Members and Features	References
***Kynurenines and associated elements***	
KYNA	[137]
KAT II	[35]
α7nAChR ^1^	[138]
***ECS***	
AEA, 2-AG	[122,123]
DAGL, MAGL	[139,140]
CB_1_R	[141]
***Common points***	
Involved in the THC-induced enhanced glutamate release	[142]
Co-localized CB_1_R and α7nAChRs mRNA	[142]

^1^ although other KYNA receptors are present in astrocytes (see Section 4.3.2.), α7nAChRs have been the most promising candidate for mediating the effects of KYNA associated with schizophrenia [61].

**Table 4:** Kynurenines and associated elements (enzymes, receptors) and members of the ECS that participate in the inflammatory mechanism of schizophrenia. The table also highlights the common points between the two systems relevant to this aspect.

Members and Features	References
***Kynurenines and associated elements***	
l-KYN, KYNA, 3-HK	[15,35,172]
KAT, IDO, KMO	[173,174,175]
GPR35 ^1^, AHR ^1^	[14,15]
***ECS***	
AEA, 2-AG	[176,177,178]
CB_2_R, CB_1_R	[19,179]
***Common points***	
Cytokine regulation, microglial activation	[19,35,175,177,178,179]
Oxidative stress	[16,17,20,180,181,182,183,184]
KYNA and endocannabinoids communicate with gut microbiome	[14,18,185,186,187]
Involvement in IBD	[18,186,188]
Common features of GPR35 and CBRs	[49,78,189,190,191]

^1^ targeted by KYNA.

**Table 5:** A summary of potential therapeutic approaches for schizophrenia by targeting the kynurenine pathway (KP) and ECS.

Approaches	References
***Kynurenine pathway***	
KAT II inhibition	[22,23,24,25,27]
IDO, TDO KMO inhibition	[7,26,263]
***ECS***	
FAAH inhibition (including CBD)	[264,265,266,267,268,269,270,271]
CB_1_R activation	[272]
CB_1_R blockade	[273,274,275]
